# Forgotten indwelling stent in a transplanted kidney: a case report

**DOI:** 10.1186/1757-1626-2-27

**Published:** 2009-01-08

**Authors:** Shaheel Bhuva, Steven J Kennish, Tze M Wah

**Affiliations:** 1 Department of Radiology, St James' University Hospital, Beckett Street, Leeds, LS9 7TF, UK

## Abstract

**• Introduction:**

Forgotten or retained ureteric stents are a well-recognised phenomenon with the potential to cause a range of complications, the most dangerous of which is obstructive nephropathy. These risks are potentially devastating when the patient has a single functioning transplanted kidney. Here we describe the case of a renal transplantation patient with a forgotten ureteric stent of 10 years, who presented with irritative bladder symptoms and was successfully managed using a multimodal urological approach with specialist advice on antibiotic prophylaxis. To the best of our knowledge this is the longest documented time period for a forgotten ureteric stent in a transplantation patient and is unusual in that obstructive nephropathy did not occur.

**• Case presentation:**

A 32-year-old man with a history of end stage renal failure of unknown aetiology received a cadaveric renal transplant in 1995. An indwelling JJ stent was placed at the time of transplant to protect the vesicoureteric anastomosis. The patient made an unremarkable recovery and initially attended regular follow up in the renal transplant clinic. He was subsequently lost to transplant clinic follow up. In 2005 at the age of 42 he was referred to a nephrologist with irritative bladder symptoms. Renal tract imaging with ultrasound and a plain film demonstrated a retained encrusted ureteric stent.

**• Conclusion:**

The removal of a retained encrusted ureteric stent always provides a urological challenge. This case demonstrates that multimodal treatment involving a combination of endourological and percutaneous techniques can be employed with success even when the patient has a heavily encrusted stent for a single functioning transplanted kidney. Involvement of a microbiologist to advise on prophylactic antibiotics is deemed especially useful, as the immunosuppressed transplant patient is at particular risk of sepsis secondary to bacteraemia as a result of the endoscopic manipulation of the colonised encrusted stent. This case also provides further evidence to highlight the potential benefits of a stent registry.

## Introduction

Ureteral stents are commonly used in the management of urological problems. In addition to their importance as an adjunct to ureteral reconstruction in renal transplant surgery [1], other indications include relief of ureteral obstruction, ensuring adequate postoperative drainage and prevention of ureteral injury during surgery. [2]

Serious complications are however associated with their use, especially when retained or forgotten. These include migration, fragmentation, encrustation and stone formation, which can lead to obstructive nephropathy, a potentially devastating consequence for a patient with a single functioning kidney. [3] Nicol et al reported a 2.2% incidence of stent complications in the renal transplant population. [1]

There are very few documented cases of forgotten ureteric stents in renal transplant patients in the medical literature, probably because of regular outpatient follow up. Here we present the case of a patient with a forgotten ureteric stent of 10 years who presented with irritative bladder symptoms and was successfully managed using a multimodal urological approach.

## Case presentation

A 32-year-old man with a history of end stage renal failure of unknown aetiology received a cadaveric renal transplant in 1995. An indwelling JJ stent was placed at the time of transplant to protect the vesicoureteric anastomosis. The patient made an unremarkable recovery and initially attended regular follow up in the renal transplant clinic. He was lost to transplant clinic follow up after this time.

**Figure 1:**
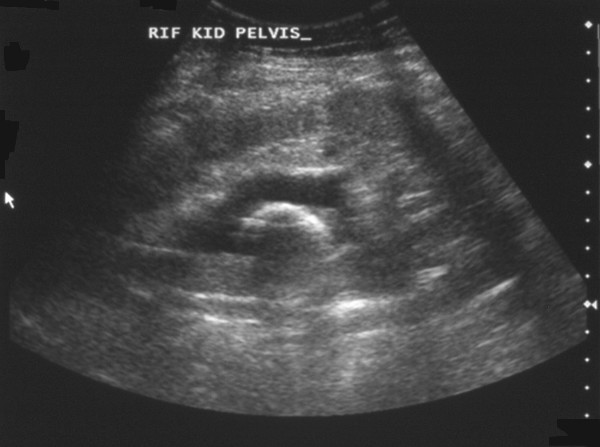
**Ultrasound scan demonstrating hyper-reflective encrusted ureteric stent**.

**Figure 2:**
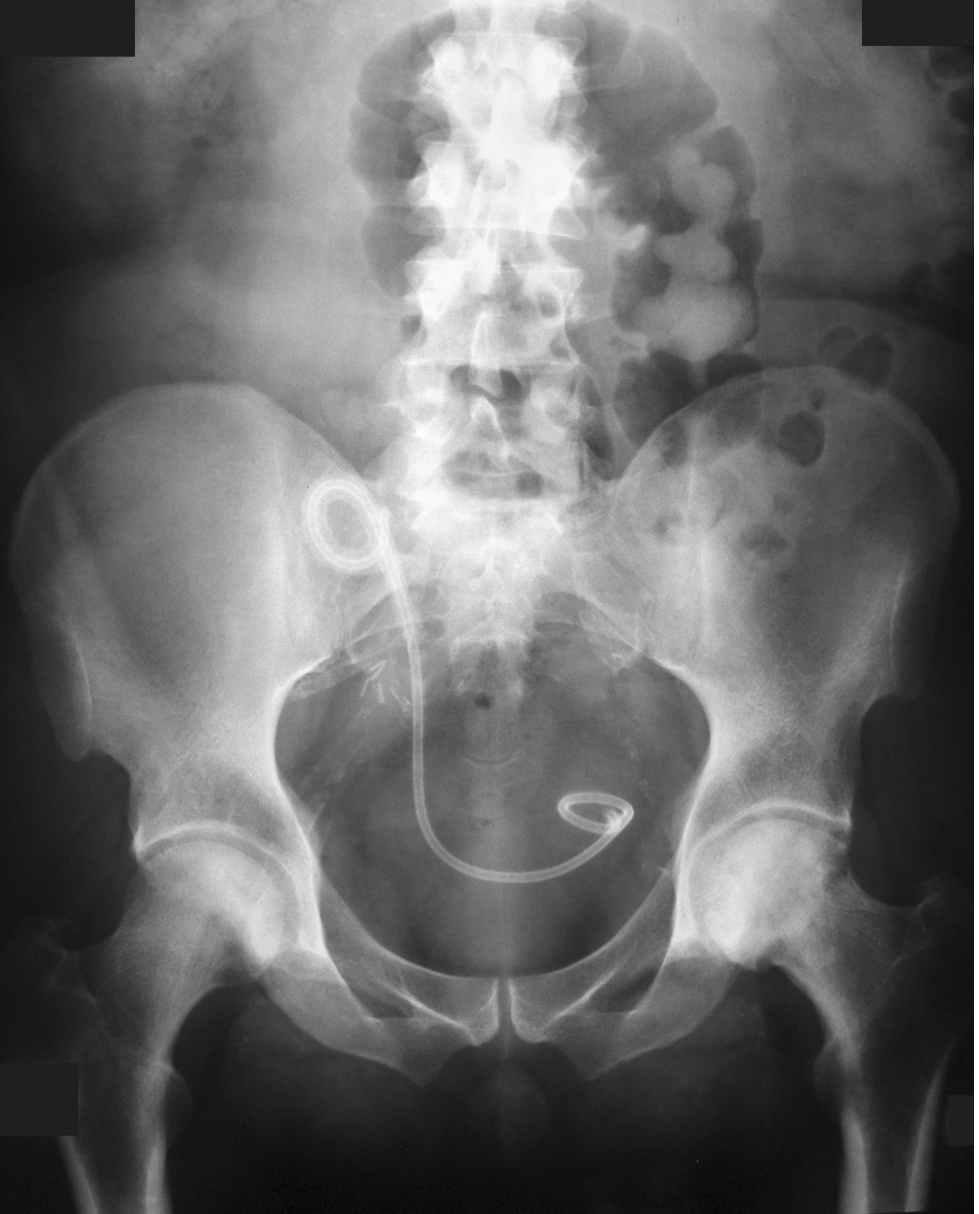
**Plain film demonstrating encrusted stent**.

In August 2005 the primary care physician expedited the patient's routine appointment with the nephrologist because of new symptoms of nocturia and poor urinary stream. An ultrasound scan was performed demonstrating moderate hydronephrosis and a calcified stent much to the surprise of the radiologist, patient and nephrologists, Figure 1. A Kidney-Ureter-Bladder (KUB) plain radiograph also demonstrated the calcified stent with bulky encrustations at the renal pelvis and intra-vesical portions, Figure 2. Biochemical assessment of renal function was satisfactory.

In September 2005, at the age of 42, he underwent cystoscopic clearance of distal encrustations. Difficulty accessing the vesicoureteric orifice and proximal stone burden rendered cystoscopic retrieval alone unrealistic. Percutaneous Nephrolithotomy (PCNL) was employed which allowed destruction of the upper ureteric stones and removal of the encrusted JJ stent in tact. A postoperative nephrostogram demonstrated no residual calculi.

The patient continued to make an excellent recovery and was relieved of his irritative bladder symptoms. A follow up KUB radiograph confirmed clearance of all calculi, and biochemical renal function tests remained satisfactory.

## Discussion

A strict definition for 'forgotten' does not exist, however many previous studies consider a period of greater than 6 months to constitute a forgotten stent. [2] It is also widely considered that a retained stent is one that is irretrievable cystoscopically and requires a further auxiliary intervention. [4,5]

Several risk factors have been identified for the development of stent encrustation. These include: a history of urolithiasis, recurrent urinary tract infections, prolonged stenting and stent composition. [3,4,6] It should be noted that the risk of stent encrustation is proportional to indwelling time and hence this risk factor is of particular importance. [4]

Our patient retained his forgotten ureteral stent for 10 years following his renal transplant, despite regular follow-up. This to the best of our knowledge is the longest documented time period for a forgotten ureteric stent in a transplantation patient. He had comfortably and unwittingly tolerated it until the development of irritative bladder symptoms, which led to him seeking medical help, and the subsequent discovery of the stent.

Challacombe et al, [7] conducted a retrospective analysis of 2085 renal transplants. 21 of the 2085 developed urolithiasis. Of note, 1 developed renal calculi after failure of removal of a stent. The overall range of presentation included pain, haematuria, oliguria/anuria, sepsis, and renal failure.

Singh et al, presented a retrospective analysis of 19 forgotten indwelling ureteric stents. [8] Renal transplantation was the indication for stenting in only one case. Overall, presentation at time of diagnosis included 7/19 with irritative bladder symptoms. Haematuria, recurrent UTI, stone formation, loin pain, renal failure and infected hydronephrosis made up the remaining 12 cases. The renal transplant patient with a forgotten stent of 1 year presented with recurrent UTI and intermittent haematuria. Within this case series is also a patient with a solitary functioning kidney stented for 10 years for mid-ureteric stone. This patient presented with advanced renal failure and hyperkalaemia, refused treatment and subsequently died.

An encrusted, retained stent presents a significant urological challenge and many previous studies have not only acknowledged the associated risk of morbity but also the necessity of a multimodal endourological approach. [2,9-14]

These modalities include Extracorporal Shockwave Lithotripsy (ESWL), Ureteroscopy +/- intracorporal retrieval (URS), and Percutaneous Nephrolithotmy (PCNL). Currently no guidelines exist for the effective management of forgotten stents. Previous reports recommend that size of stone burden and site of encrustation determine the specific endourologic approach, and that the distal ureteral segment should be treated prior to any other part of the stent. [11,13]

With particular reference to the use of PCNL, Lam and Gupta [13], suggested this should be reserved for a large proximal stone burden or when ESWL had failed to manage the proximal stent encrustations. Bultitude et al, considered ureteroscopic intervention with adjunctive ESWL to be efficacious with PCNL as a secondary treatment. [12]

Aravantinos et al, identified that even with successful stone fragmentation at the proximal end of the stent using ESWL, intraluminal encrustations can limit straightening and therefore prevent uncoiling of the calcified proximal curl. [2] Thus a supplementary intervention is required. In addition if it is difficult to pass a ureteroscope into the ureter, which already contains an encrusted stent, PCNL is a reasonable approach.

Once the distal end of the stent is cleared of stone burden using a ureteroscopic approach, management of a stone covered encrusted proximal stent using PCNL is reasonable. Though a more invasive technique, it necessitates fewer subsequent interventions, allowing for fragment retrieval and stent and stone clearance at the same time (an all-in-one method). [9] In addition, a nephrostomy tube can be placed guaranteeing adequate drainage, enabling postoperative imaging exploration, and avoiding ureteral restenting. [2]

A particular concern with the immunosuppressed transplant patient is the risk of sepsis secondary to bacteraemia as a result of endoscopic manipulation of colonised stents and stones. This can arise despite sterile urinary cultures and can be devastating in compromised patients. [9] The advice of a microbiologist regarding prophylactic measures is invaluable, though any risk of sepsis must be communicated to the patient adequately for the purposes of consent prior to the procedure.

## Conclusion

The forgotten ureteric stent can cause considerable morbidity to patients and pose challenging urological problems. The stakes are far greater when a single functioning kidney is at risk in an immunosuppressed patient. This case demonstrates that a multimodal endourological and percutaneous technique can safely remove a heavily encrusted stent. Specialist microbiological advice for antibiotic prophylaxis to prevent sepsis in an immunosuppressed patient is advocated. The use of a stent registry and patient notification card has been widely recommended to prevent this situation from arising. [14] Certainly the importance of regular follow up and clear patient education as well as good communication between the transplant surgeons and nephrologists must be emphasized to prevent serious complications.

## Consent

Written informed consent was obtained from the patient for publication of this case report and accompanying images. A copy of the written consent is available for review by the Editor-in-Chief of this journal.

Dr Shaheel Bhuva

Dr Steven Kennish

Dr Tze Wah

## Competing interests

The authors (Dr Shaheel Bhuva, Dr Steven Kennish and Dr Tze Wah) declare that they have no competing interests.

## Authors' contributions

TW was involved with the initial case management and acquired relevant images. SB undertook a literature review. The report was written by SB and SK and edited by TW.
